# Supplementary material for: Atypical plant homeodomain of UBR7 functions as an H2BK120Ub ligase and breast tumor suppressor
Source: Nat Commun. 2019 Mar 28;10:1398. doi: 10.1038/s41467-019-08986-5 (PMC6438984; doi:10.1038/s41467-019-08986-5)
Supplement: Supplementary file 1 — Supplementary Information [file 41467_2019_8986_MOESM1_ESM.pdf]

Atypical Plant Homeodomain of UBR7 Functions as an H2BK120Ub Ligase and Breast Tumor Suppressor

Adhikary et al., 2019

Supplementary Information

Supplementary Figures

Supplementary Tables

Supplementary Figure 1

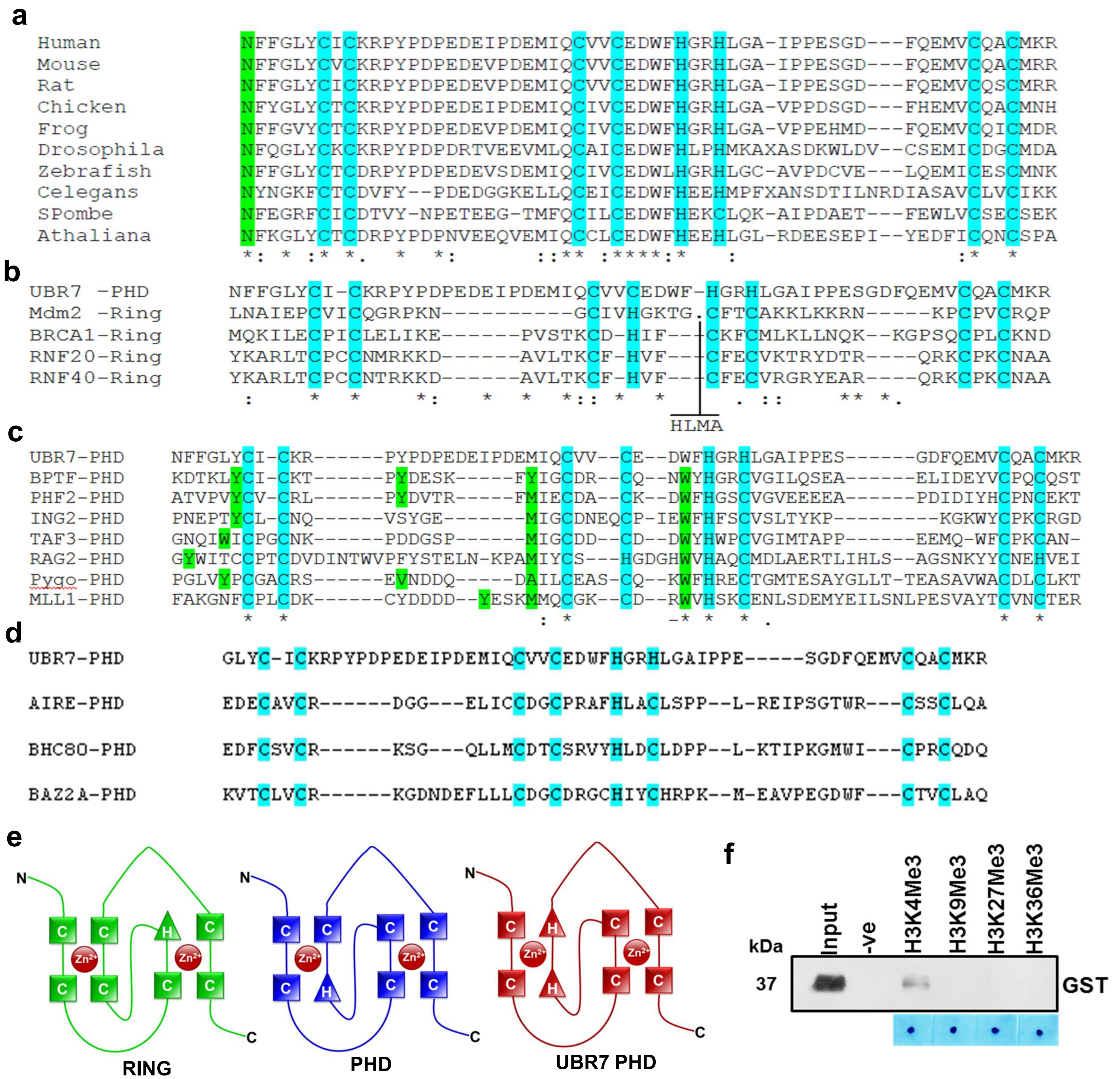

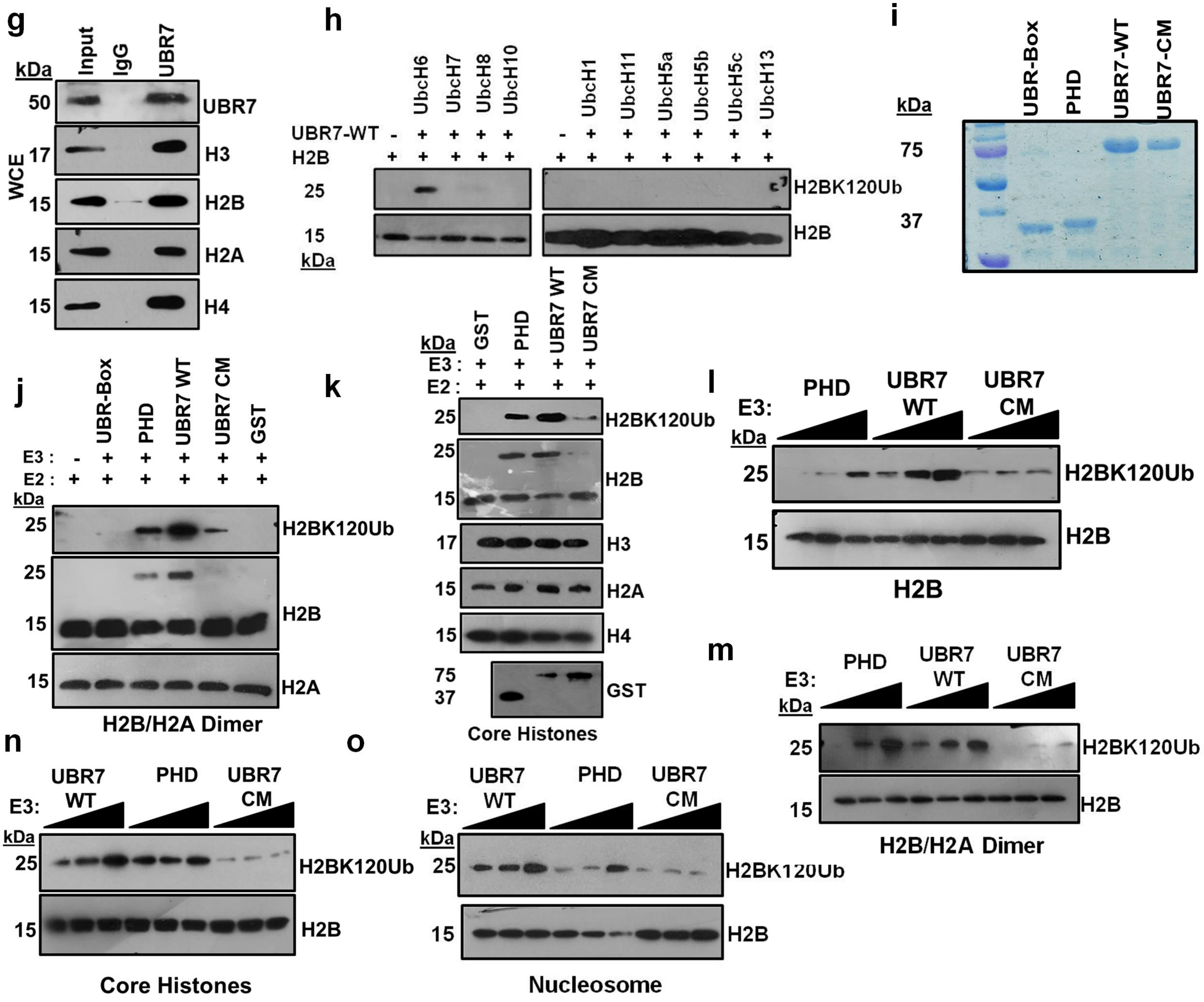

Supplementary Figure 1: UBR7 alignment and its E3 ubiquitin ligase activity for H2BK120.
a-d Sequence alignment **(a)** showing that UBR7 is conserved across species. A comparison of the PHD finger of UBR7 with other canonical RING finger proteins **(b)** and PHD finger proteins, that binds to H3K4Me3 **(c)** or unmodified histone H3 **(d)** is shown**. e** Cross brace topology depicting the organization in canonical RING, PHD and UBR7 PHD. **f** Peptide pull-down assay with the GST-PHD of UBR7 and H3 trimethylated peptides. **g** Co-Immunoprecipitation of UBR7 from MCF10A cells indicating its interaction with core histones. **h** *In-vitro* ubiquitination assay, scanning a range of E2's to identify the corresponding E2 for UBR7. The results show that UbcH6 is the E2 for UBR7. **i** Coomassie gel profile of proteins used in all *in-vitro* assays. **j and k** *In-vitro* ubiquitination assay with H2A/H2B dimer **(j)** or core histone octamer **(k)**. **l-o** *In-vitro* ubiquitination assay using increasing concentration of UBR7-PHD, UBR7-WT or UBR7-CM with recombinant H2B **(l)**, or H2A/H2B dimer **(m)** core octamer **(n)**, or purified nucleosomes from HeLa cells **(o).**

Supplementary Figure 2

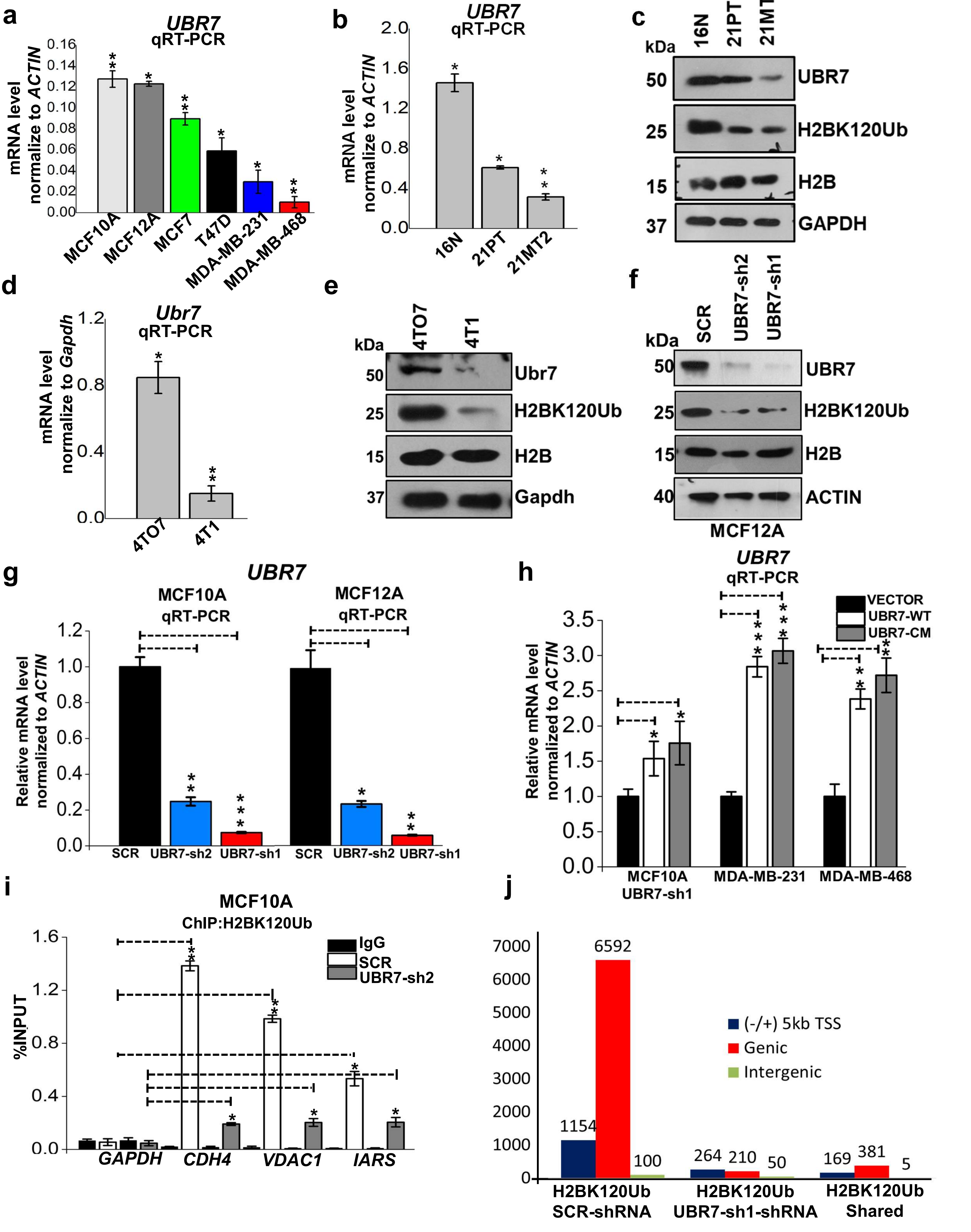

Supplementary Figure 2: UBR7 expression analysis in breast cancer cells.

a Expression of *UBR7* according to qRT-PCR analysis in different normal breast and breast cancer cell lines. Error bars indicate standard deviation (s.d.); n=3 technical replicates of a representative experiment (out of three experiments). **b-e** The expression of *UBR7* was monitored by qRT-PCR **(b and d)** and immunoblots **(c and e)** in human and murine breast cancer cell lines. Here 16N is normal, 21-PT is a benign tumor and 21-MT2 is a metastatic tumor in human **(b and c)** and 4TO7 is benign and 4T1 is breast metastatic tumor cell line in mouse **(d and e)**. GAPDH was used as a loading control for the immunoblots. **f** Immunoblots of UBR7, H2BK120Ub, H2B and ACTIN expression in MCF12A cells. ACTIN was used as loading controls**. g** qRT-PCR analysis showing the UBR7shRNAs (UBR7-sh1 and sh2) knockdown efficiency in MCF10A and MCF12A cells. **h** qRT-PCR analysis showing wild-type (UBR7-WT) and catalytic-mutant (UBR7-CM) UBR7 over-expression in MCF10A UBR7-sh1**,** MDA-MB-231, and MDA-MB-468 cells**. i** H2BK120Ub ChIP in MCF10A cells expressing scrambled (SCR) or UBR7-sh2 shRNA. *GAPDH* was used as a negative control. **j** Enrichment of H2BK120Ub across genomic loci in MCF10A cells expressing scrambled (SCR) or UBR7-sh1 shRNA. *P*-values were calculated using two-tailed *t*-tests. In panels **a**, **b**, **d**, **g**, **h** and **i** error bars indicate standard deviation (s.d.); n=3 technical replicates of a representative experiment (out of three experiments). *$P<0.05$; **$P<0.001$; ***$P<0.0001$.

Supplementary Figure 3

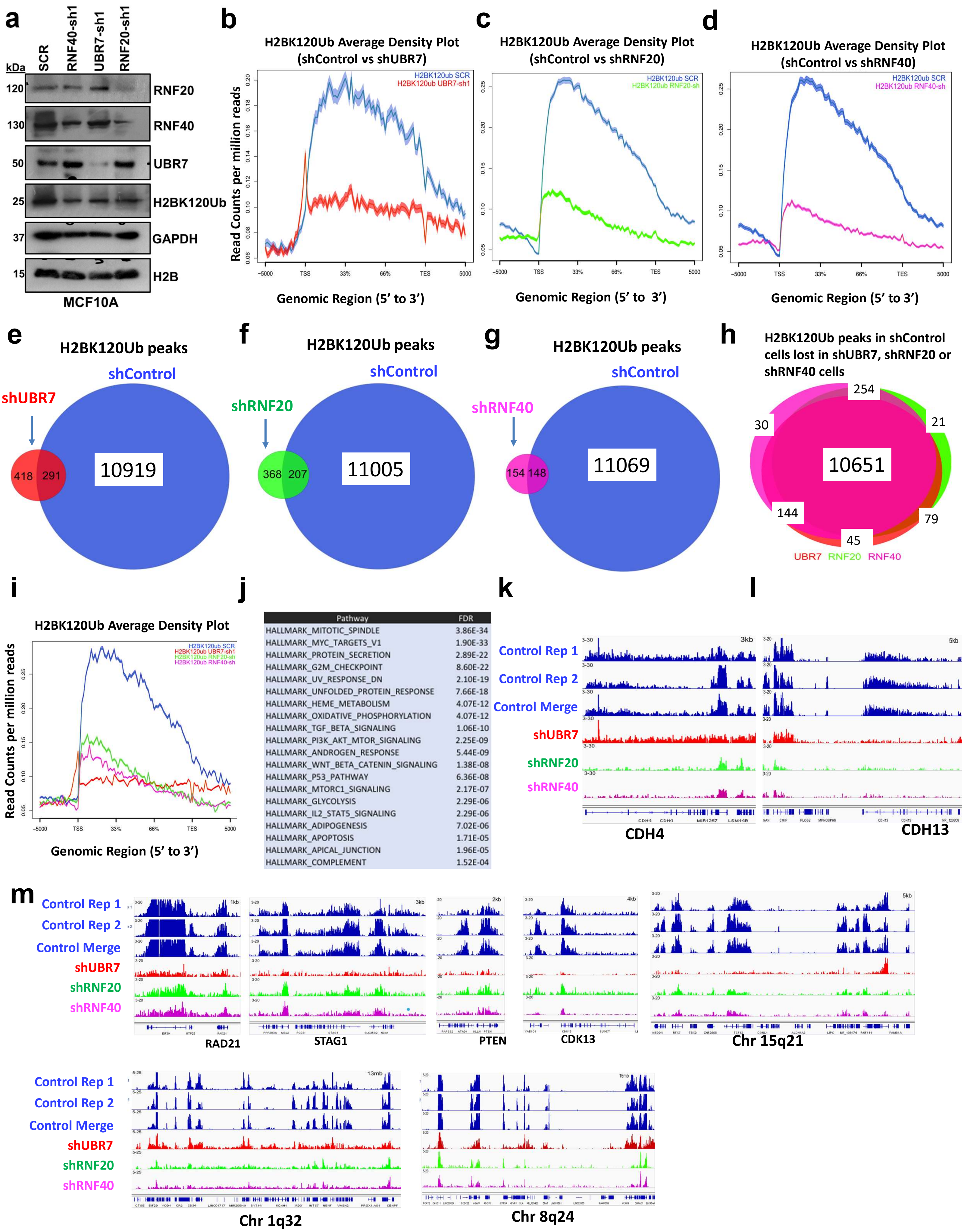

Supplementary Figure 3: Relationship between H2BK120Ub sites lost in UBR7-deficient, RNF20-deficient and RNF40-deficient cells.

a Immunoblot for RNF20, RNF40, UBR7, H2BK120Ub, GAPDH and H2B in MCF10A cells harbouring UBR7, RNF20 and RNF40 shRNAs. GAPDH was used as a loading control. **b-d** Average genebody density plot for H2BK120Ub binding sites (randomly sampled to ~10 million uniquely mapped reads) in **(b)** replicate1 Control (SCR) and UBR7 shRNA-1 expressing MCF10A cells **(c)** replicate2 Control (SCR) and RNF20 shRNA expressing MCF10A cell and **(d)** replicate2 Control (SCR) and RNF40 shRNA expressing MCF10A cells. **e-g** Venn diagram showing overlap of total H2BK120Ub binding sites in merged Control (SCR) and UBR7 shRNA-1 **(e)**, RNF20 shRNA **(f)**, or RNF40 shRNA (**g**) expressing MCF10A cells. **h** Venn diagram showing overlap of total H2BK120Ub binding sites in merged Control (SCR) shRNA expressing MCF10A cells that are lost upon knockdown of UBR7, RNF20 or RNF40. **i** Average genebody density plot for H2BK120Ub binding sites in merged Control (SCR) and UBR7, RNF20 or RNF40 shRNA expressing MCF10A cells. **j** Gene set enrichment analysis using significantly enriched HALLMARK showing pathways (FDR < 1e-3) enriched for H2BK120Ub binding sites in merged Control (SCR) shRNA expressing MCF10A cells that are commonly lost upon knockdown of UBR7, RNF20 or RNF40in genes that lose H2BK120Ub mark in UBR7, RNF20 and RNF40 shRNA containing cells. **k-m** IGV view of H2BK120Ub ChIP-seq tracks on the *CDH4* **(k)**, *CDH13* **(l)** and other regions (*RAD21, STAG1, PTEN, CDK13*, Chr1q32, Chr15q21 and Chr8q24) **(m)** from replicate1, replicate2 and merged Control (SCR), UBR7, RNF20 or RNF40 shRNA expressing MCF10A cells. Regions in m are shown to demonstrate data quality as well as peaks that are lost specifically in UBR7 knockdown (RAD21, STAG1, PTEN, multiple genes on Chr15q21) versus those specific to RNF20/40 knockdown cells (multiple genes on Chr1q32 and Chr8q24).

Supplementary Figure 4

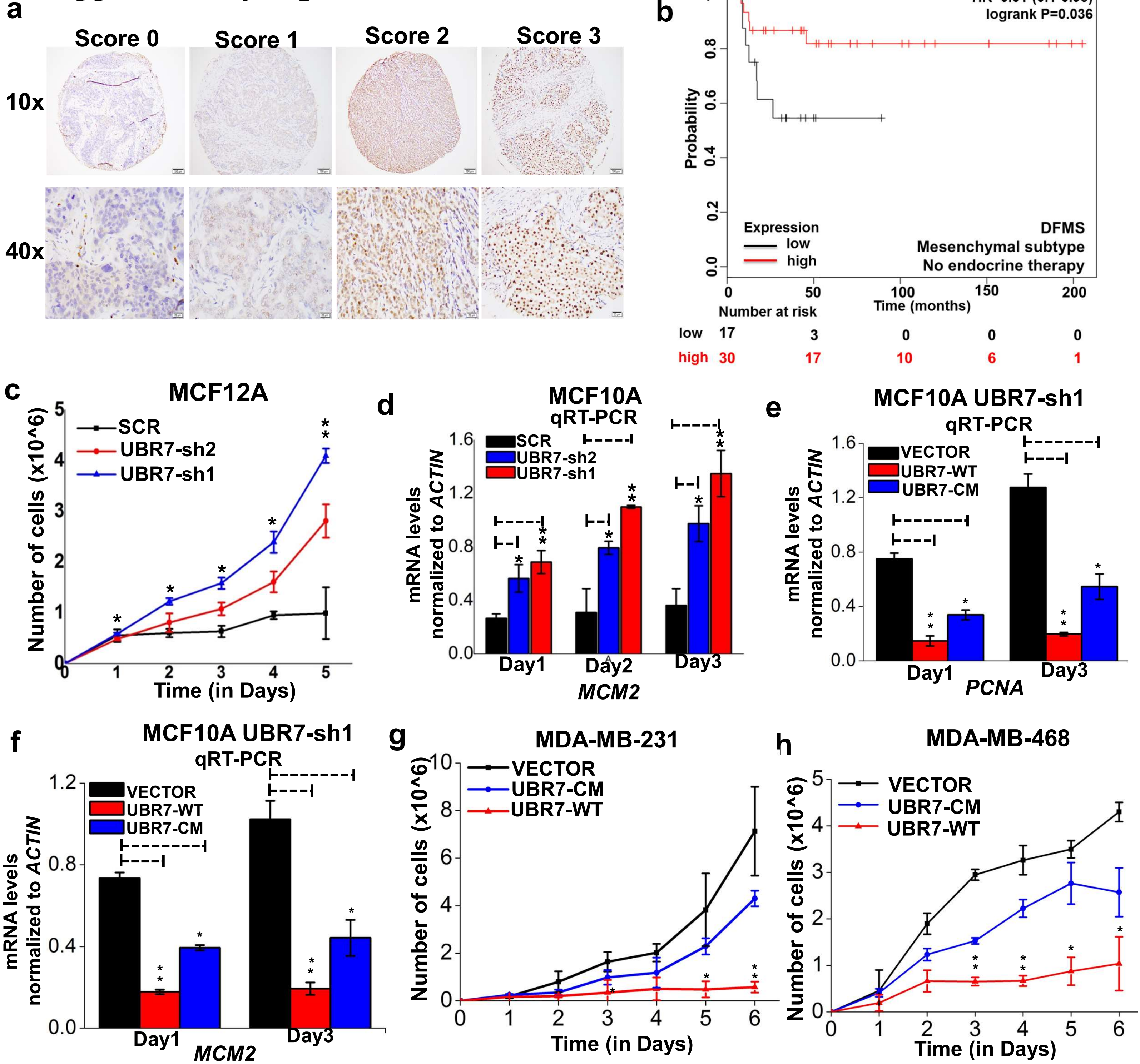

Supplementary Figure 4: UBR7 loss promotes proliferation of breast cancer.

a Immunohistochemical analysis of human breast cancer tumor samples. The representative figure shows the basis for assigning scores to the TMA. Scale bar indicates 20µm. **b** Kaplan-Meier survival analysis of patients with low (black) and high (red) UBR7 expression. High expression of UBR7 was significantly correlated with high survival rates of patients with a mesenchymal/basal subtype of breast cancer (*P* value =0.036; hazard ratio (HR) = 0.31). The analysis was performed using an online analysis tool. **c** Proliferation of cultured MCF12A cells expressing scrambled (SCR) or UBR7 shRNAs. **d-f** qRT-PCR analysis of *MCM2* **(d)** in MCF10A cells expressing scrambled (SCR) or *UBR7* shRNAs and *PCNA* **(e)** or *MCM2* **(f)** expression in MCF10A UBR7-sh1cells expressing a vector (VECTOR), wild type (UBR7-WT) or catalytic mutant (UBR7-CM) in a time-

dependent manner. **g and h** Proliferation of cultured MDA-MB-231 **(g)** and MDA-MB-468 **(h)** cells expressing a vector (VECTOR), wild type (UBR7-WT) or catalytic mutant (UBR7-CM). In all panels error bars indicate standard deviation (s.d.); n=3 technical replicates of a representative experiment (out of three experiments). *P*-values were calculated using two-tailed *t*-tests. *$P<0.05$; **$P<0.001$; ***$P<0.0001$.

Supplementary Figure 5

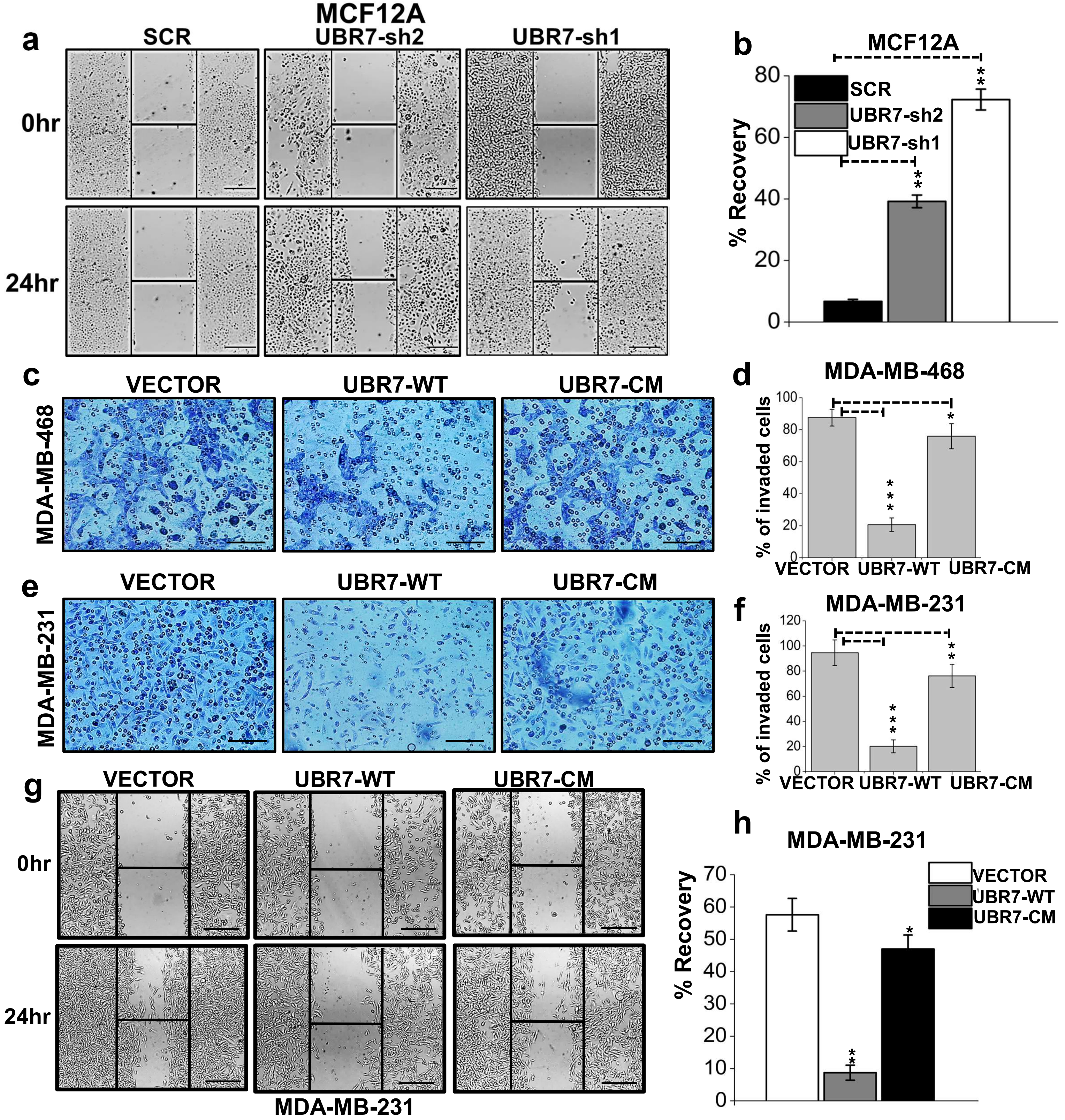

Supplementary Figure 5: UBR7 suppresses cellular invasion and migration in triple-negative breast cancer cells.

a and b Wound healing/migration rate for MCF12A cells expressing scrambled (SCR) or UBR7 shRNAs was monitored. **c-f** Invaded MDA-MB-468 **(c and d)** and MDA-MB-231 **(e and f)** cells expressing wild-type (UBR7-WT) or catalytic-mutant (UBR7-CM) in a Matrigel Chamber were photographed and quantitated. **g and h** Wound healing/migration rate for MDA-MB-231 cells expressing wild-type (WT) or catalytic-mutant (CM) UBR7 was monitored. In all panels scale bar indicates 10µm. In all panels error bars indicate standard deviation (s.d.); n=3 technical replicates

of a representative experiment (out of three experiments). *P*-values were calculated using two-tailed *t-tests*. *$P<0.05$; **$P<0.001$; ***$P<0.0001$.

Supplementary Figure 6

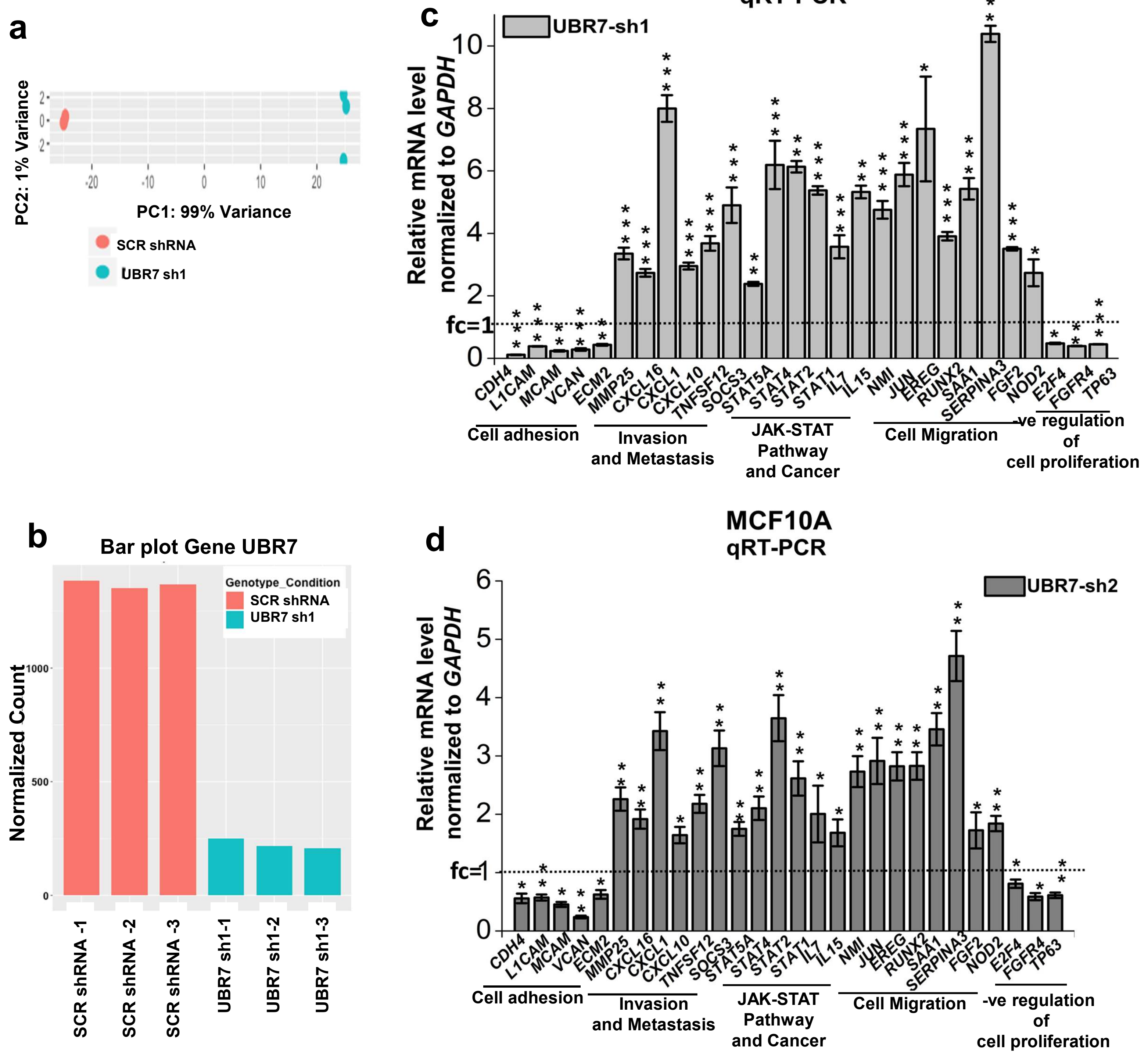

Supplementary Figure 6: Analysis of RNA-seq data after *UBR7* knock-down in MCF10A cells.

a PCA plot of RNA-seq data for MCF10A cells expressing scrambled (SCR) or UBR7-sh1shRNAs. **b** Bar plot of normalized UBR7 counts in three replicates showing consistent UBR7 knockdown in UBR7-sh1 cells compared with control cells. **c and d** qRT-PCR analysis of differentially expressed genes upon *UBR7* knockdown in MCF10A cells harboring UBR7-sh1 **(c)** and UBR7-sh2 **(d)** . In all panels error bars indicate standard deviation (s.d.); n=3 technical replicates of a representative experiment (out of three experiments). *P*-values were calculated using two-tailed *t*-tests. *P<0.05; **P<0.001; ***P<0.0001.

Supplementary Figure 7

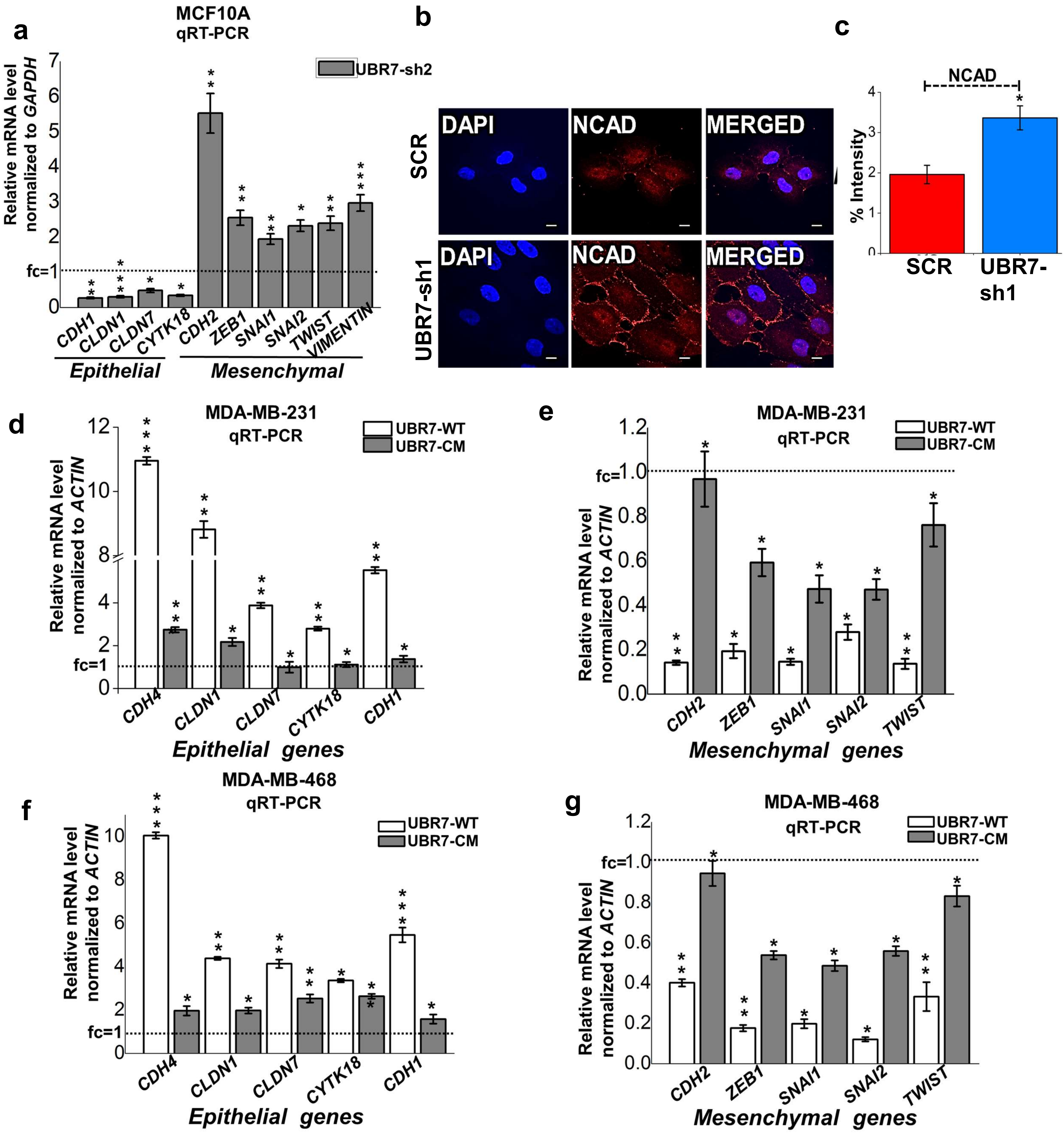

Supplementary Figure 7: UBR7 inhibits EMT by modulating gene expression governing this process.

a qRT-PCR analysis of EMT signature genes upon *UBR7* knockdown (with UBR7-sh2) in MCF10A cells. **b and c** Immunofluorescent studies demonstrating expression of NCAD after *UBR7* knockdown (UBR7-sh1) in MCF10A cells. The percent intensity is shown. Scale bar indicates 100μm. **d-g** qRT-PCR analysis of epithelial **(d and f)** and mesenchymal **(e and g)**

genes expression in MDA-MB-231 **(d and e)** and MDA-MB-468 **(f and g)** cells expressing wild-type (UBR7-WT) and Catalytic-mutant (UBR7-CM). In all panels error bars indicate standard deviation (s.d.); n=3 technical replicates of a representative experiment (out of three experiments). *P*-values were calculated using two-tailed *t*-tests. $^{*}P<0.05$; $^{**}P<0.001$; $^{***}P<0.0001$.

Supplementary Figure 8

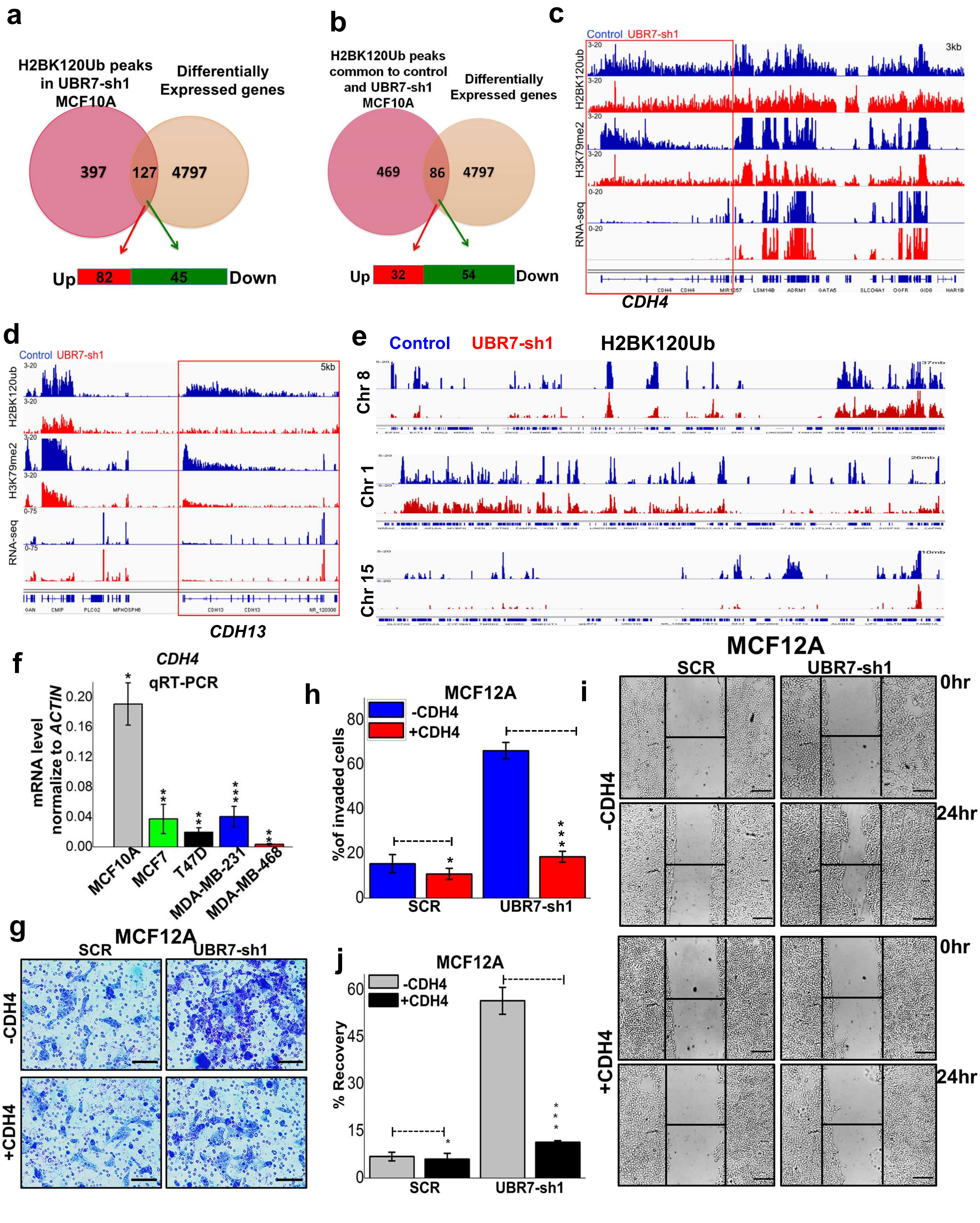

Supplementary Figure 8: UBR7 regulates cell-cell adhesion genes such as *CDH4* and *CDH13* expression there by inhibiting EMT.

a and b Venn diagrams showing overlap of H2BK120Ub UBR7-sh1shRNA **(a)** or shared **(b)** and differentially regulated genes after *UBR7* knockdown. **c-e** IGV view of H2BK120Ub and H3K79Me2 ChIP-seq signals and RNA-seq signals in the *CDH4* and surrounding locus **(c)**, *CDH13* and surrounding locus **(d)** gene and other genes on Chr1, Chr15 and Chr8 loci **(e)** in scrambled (SCR) or UBR7-sh1 shRNA expressing cells. **f** qRT-PCR analysis showing the expression of *CDH4* across different breast normal and cancer cell lines. **g and h** Invasion of MCF12A cells overexpressing CDH4 in the presence (SCR) and absence (UBR7-sh1) of UBR7 in a matrigel chamber was photographed and quantitated. **i and j** Wound healing by MCF12A cells overexpressing CDH4 in the presence (SCR) and absence (UBR7-sh1) of UBR7was photographed and percent recovery was measured over time. In panels **g** and **i** scale bar indicates 10µm. In panels **f, h** and **j** error bars indicate standard deviation (s.d.); n=3 technical replicates of a representative experiment (out of three experiments). *P*-values were calculated using two-tailed *t*-tests. *$P<0.05$; **$P<0.001$; ***$P<0.0001$.

Supplementary Figure 9

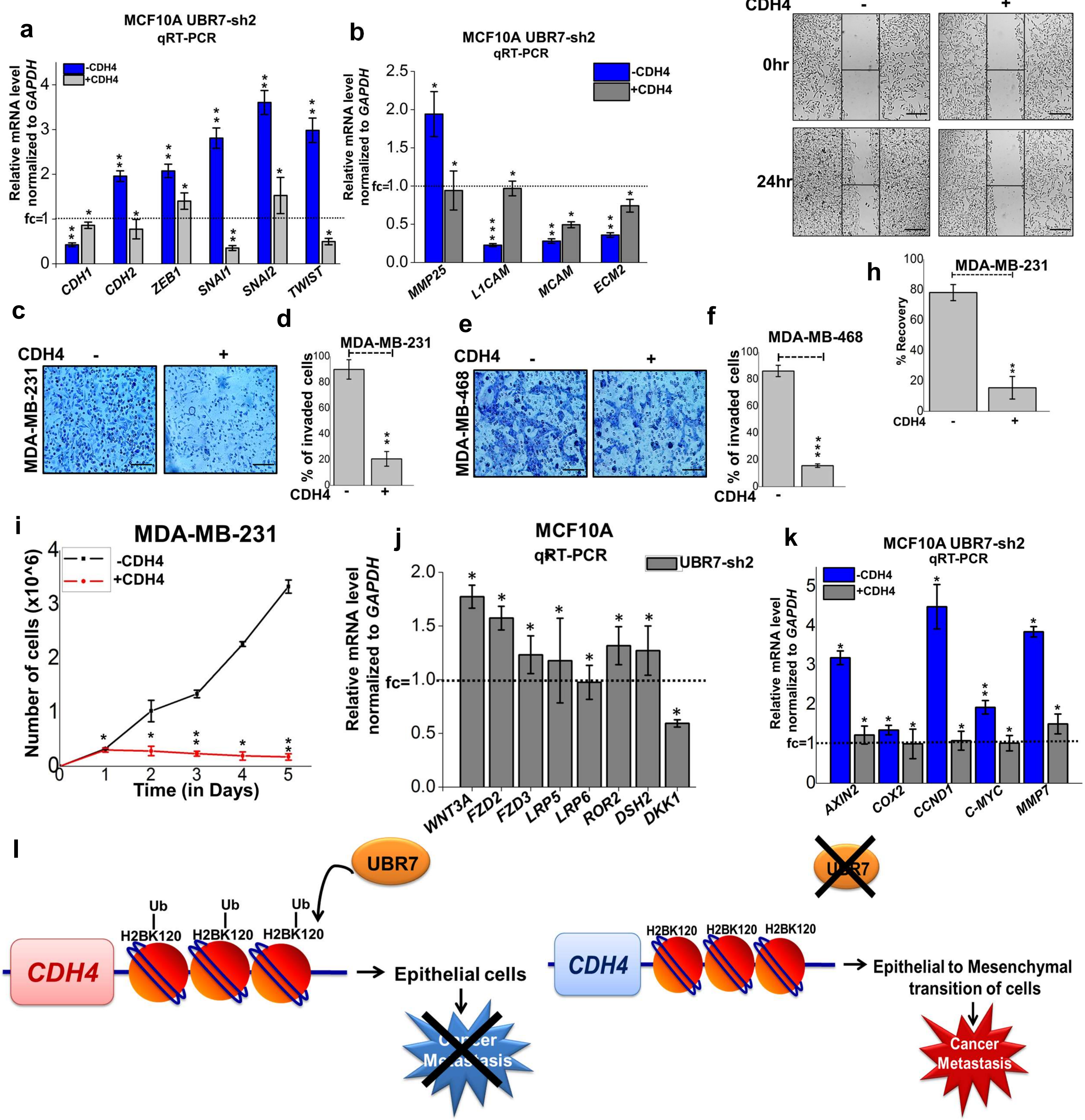

Supplementary Figure 9: CDH4 overexpression partially rescues phenotypes driven by UBR7 loss and UBR7 loss leads to activation of the Wnt/β-Catenin pathway.

a and b qRT-PCR analysis of EMT signature genes **(a)** and cell adhesion-related genes **(b)** upon CDH4 overexpression in *UBR7* knockdown (UBR7-sh2) MCF10A cells. **c-f** Invasion assay of MDA-MB-231 **(c and d)** and MDA-MB-468 **(e and f)** expressing CDH4 that were photographed and counted. **g and h** Wound healing by MDA-MB-231 cells expressing CDH4 was photographed and the percent recovery was measured over time. **i** Proliferation of cultured MDA-MB-231 cells

expressing CDH4. **j** qRT-PCR analysis of key regulators of the Wnt/β-Catenin signaling pathway upon loss of UBR7 (UBR7-sh2). **k** qRT-PCR analysis of β-Catenin target genes expression upon CDH4 overexpression in UBR7-sh2 MCF10A cells. In panels **c, e** and **g** scale bar indicates 10µm. In panels **a, b, d, f, h-k** error bars indicate standard deviation (s.d.); n=3 technical replicates of a representative experiment (out of three experiments). *P*-values were calculated using two-tailed *t*-tests. $^*P<0.05$; $^{**}P<0.001$; $^{***}P<0.0001$. **l** Schematic of the mode of action of UBR7 in suppressing breast cancer metastasis.

Supplementary Figure 10

Figure 1

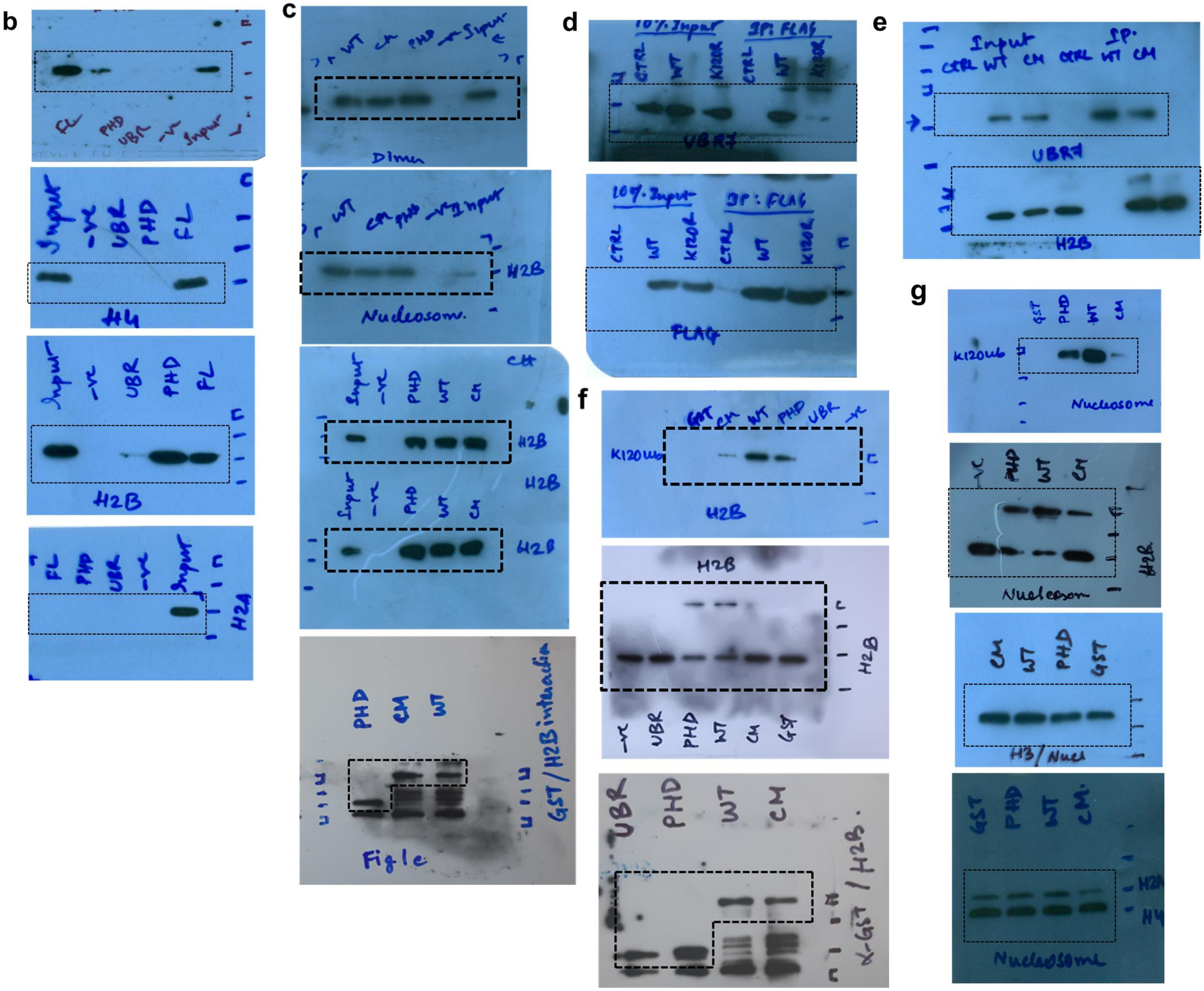

Figure 2

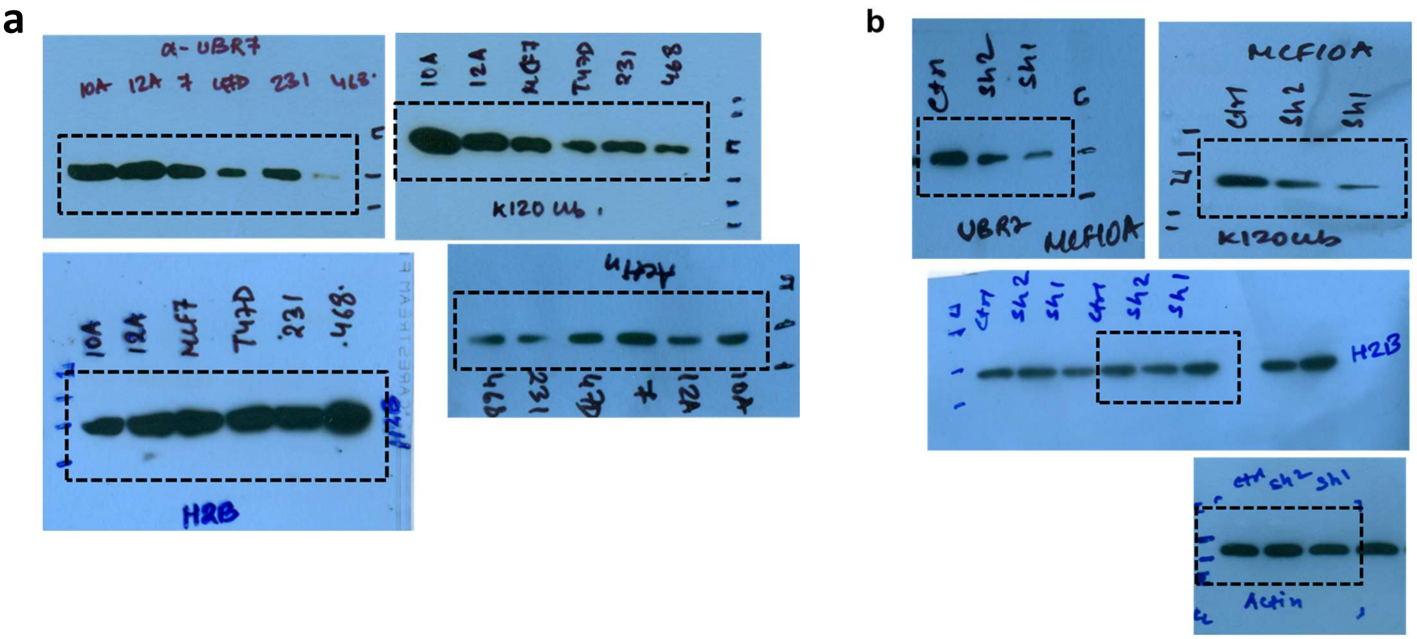

Figure 2

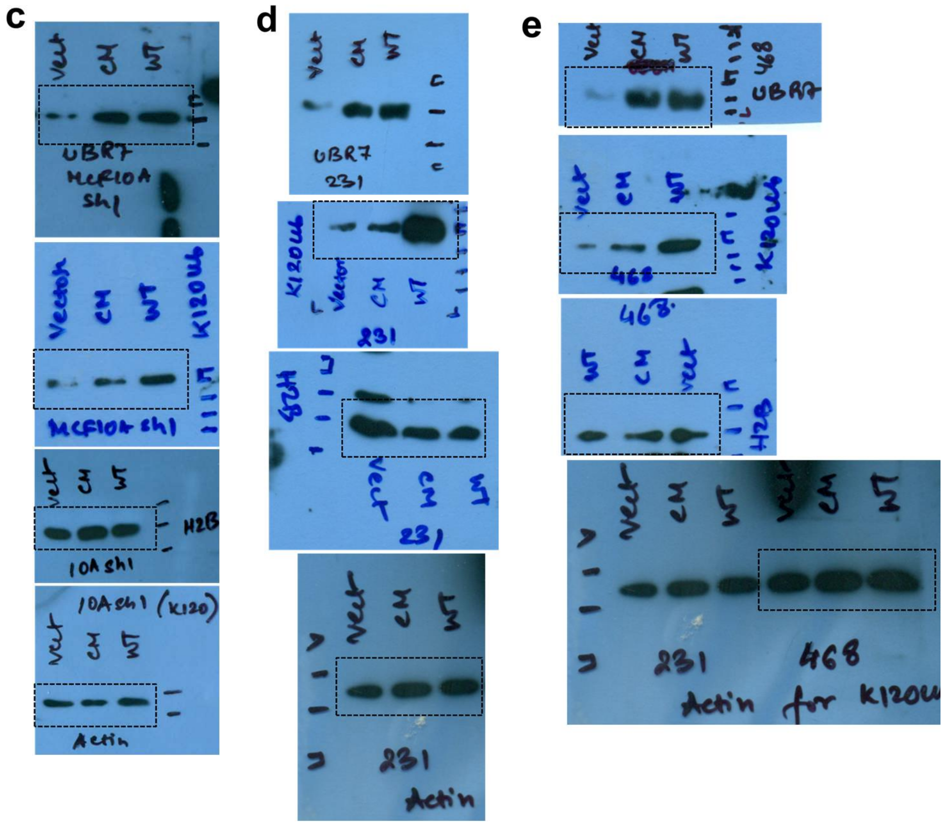

Figure 3

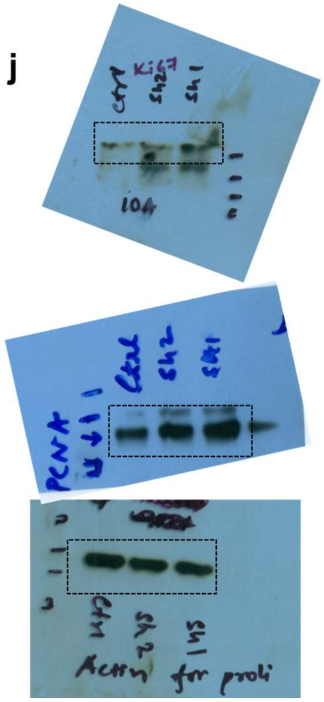

Figure 5

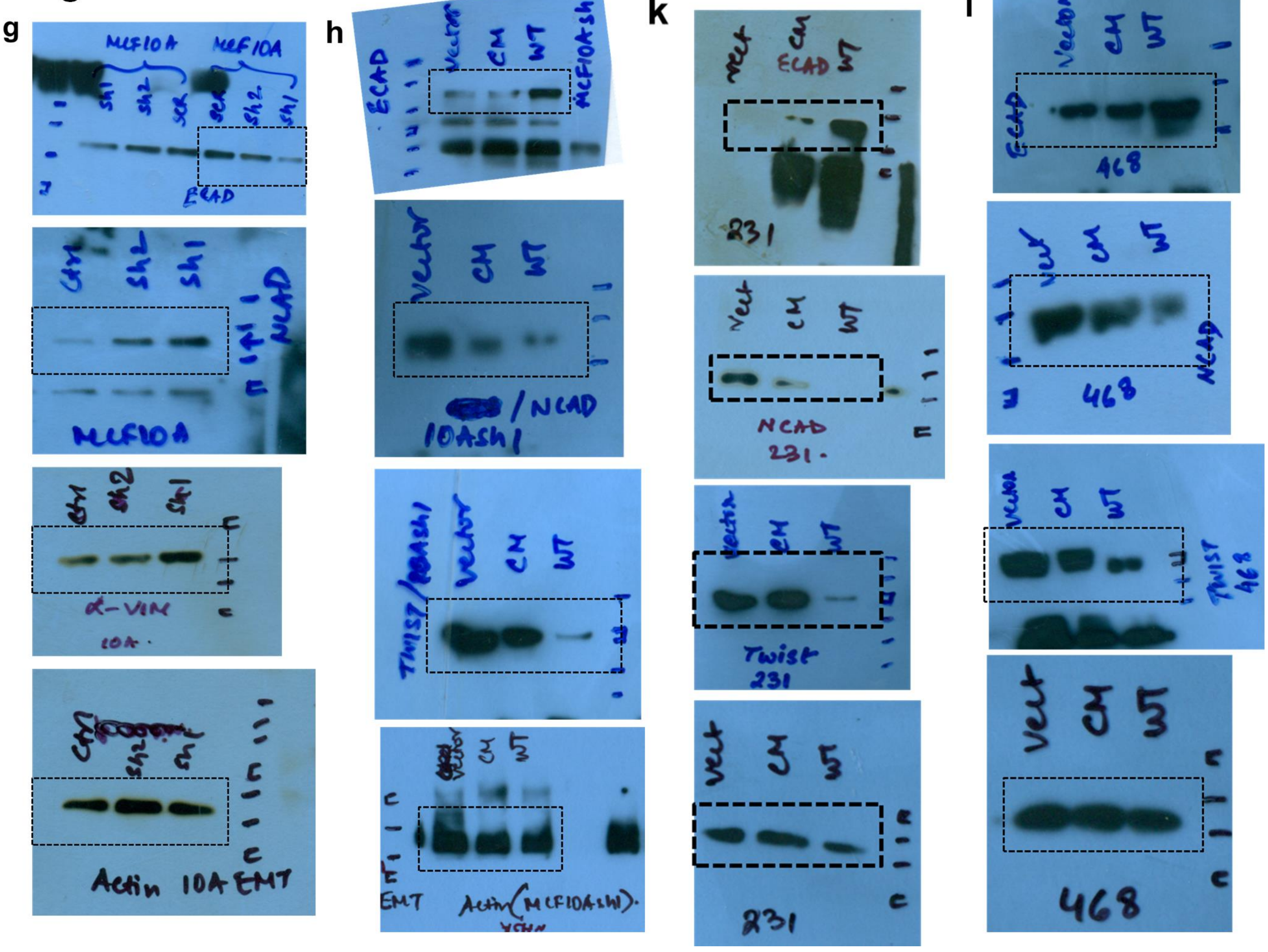

Figure 6

c

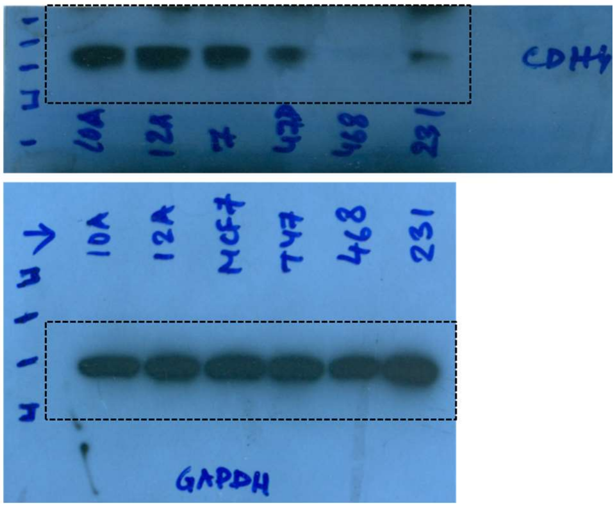

Figure 7

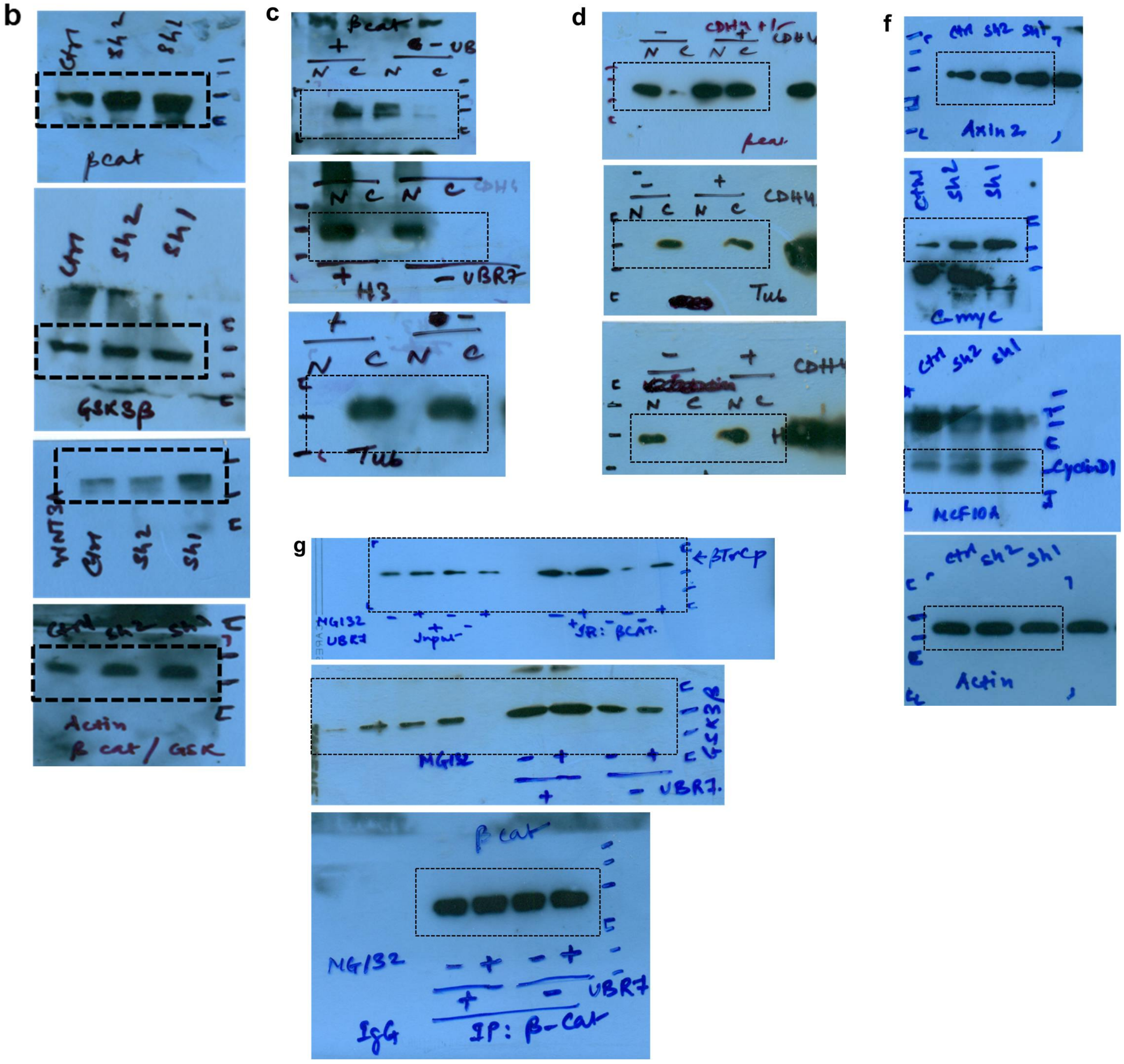

Supplementary Figure 1

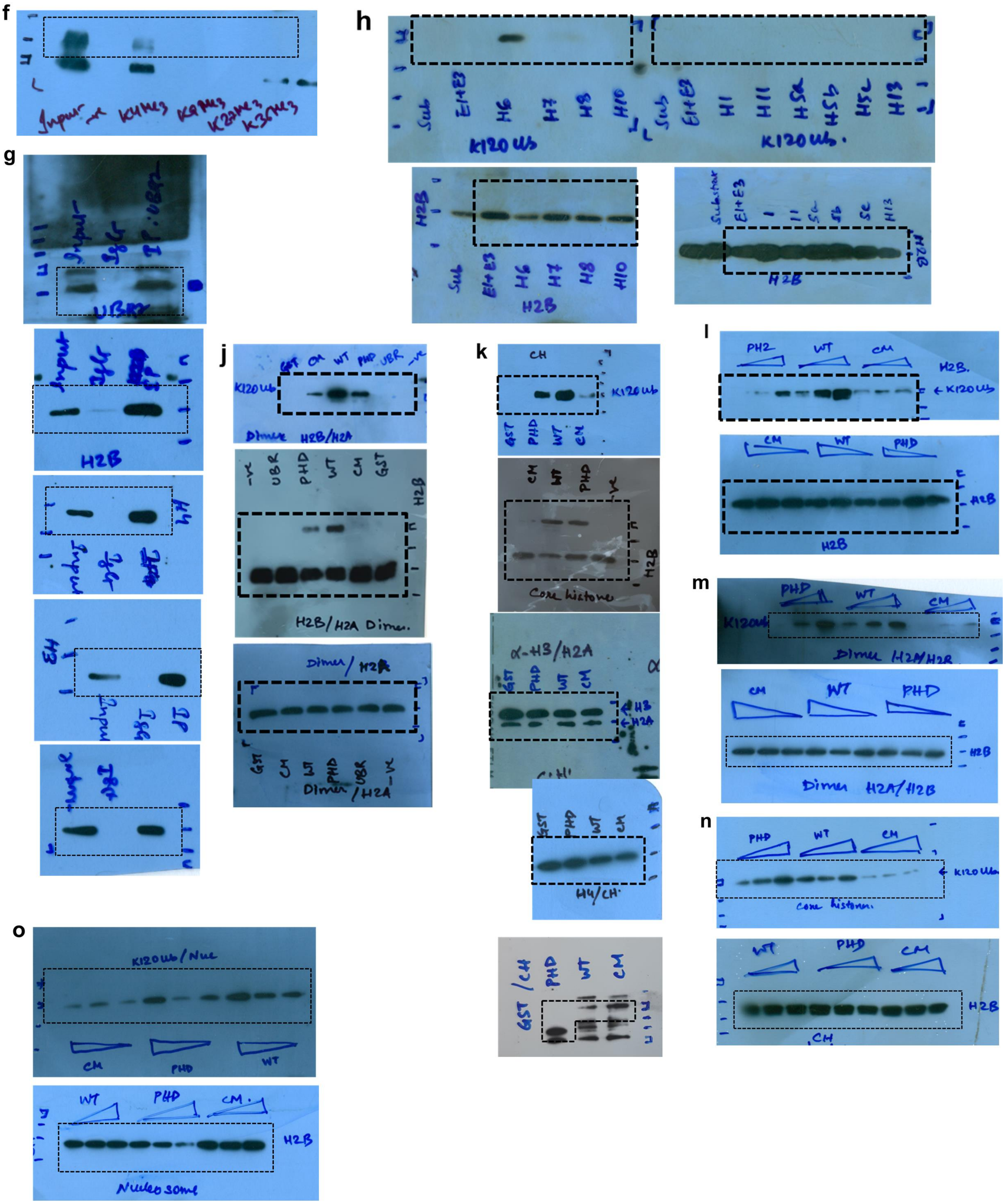

Supplementary Figure 2

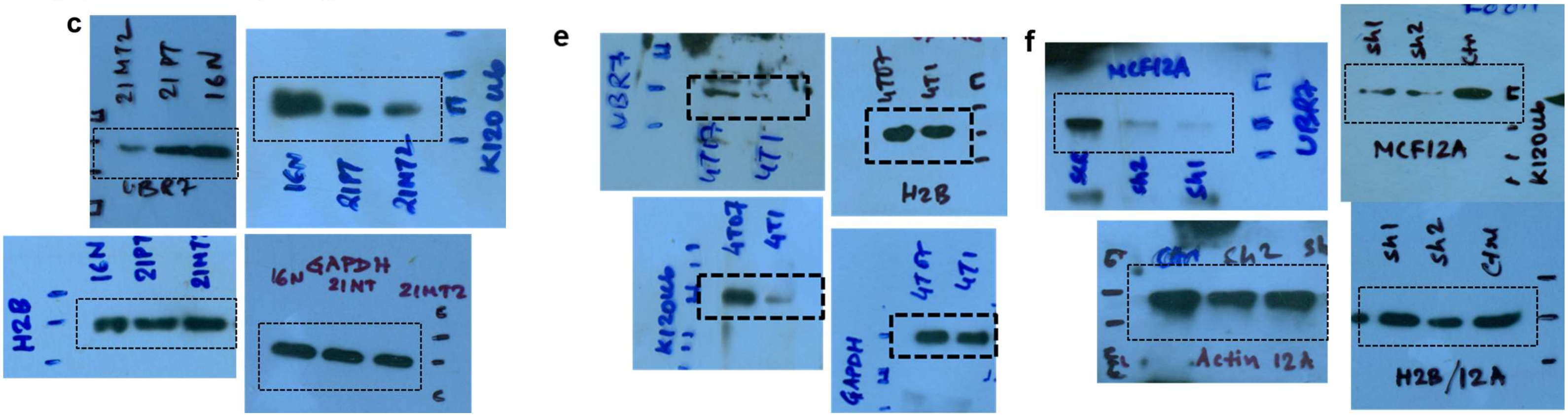

Supplementary Figure 3

a

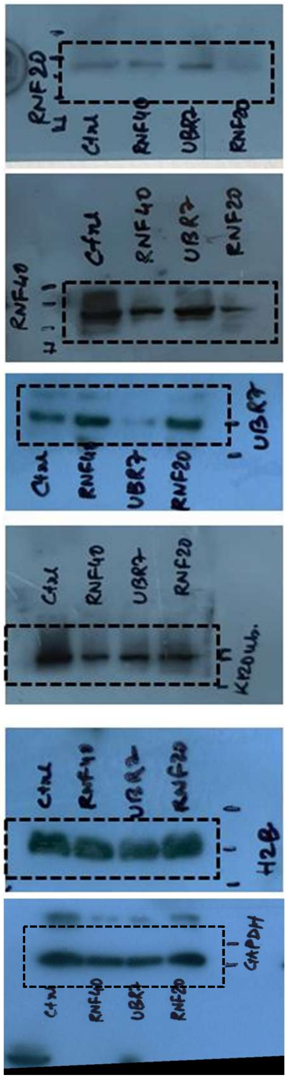

Supplementary Figure 10: Uncropped version of all blots used in the manuscript: Images of all western blot data used in figures of this manuscript.

Supplementary Table 1: List of primers used for qRT-PCR and ChIP qPCR

Gene Name	FORWARD PRIMERS (5'-3')	REVERSE PRIMER (5'-3')
UBR7	CCAGAACAGGGAAAGGATGA	TCCTGAAGTTTGCAGCCAG
SOCS3	GACTTCGATTCGGGACCAG	CTCTTGGAGCTGAAGGTCTTG
NMI	TCAAACCTGCCATAGAGTTACTG	CATCTTCTCCACCTCCATTC
STAT2	TTCTCGAAACACCTGTGGAG	TTCTGCTCTTTGGTCTGATGG
STAT5A	GAAAACATATGACCGCTGCC	CTCATTCTCTGTGTCCTGCG
STAT4	GACATTCCCAAAGACAAAGCC	CTCTCAACACCGCATACACA
L1CAM	AGGTCACTATCGGCTACTCTG	TGGATATGTCTCTTGCTGTGC
VCAN	TCCCTGCAATTACCATCTCAC	TCTTCCATTTCCTAAGCACCG
MCAM	GTCACCGTCCCTGTTTTCTAC	GTTGTCTCTTCCTCTGCCTC
ECM2	CCATGCTTACCCAGATACCAC	GAATGCTTTTGGACCTATGCC
E2F4	GTTTCTACACCTCCACCTCTG	GTCCTTGCTATCAGTCCCAG
TP63	CCCTCACTCCTACAACCATTC	CGCTAAGAAACTGACAATGCTG
FGFR4	AATGTGCTGGTGACTGAGG	ATAGCAGGATCCCAAAAGACC
MMP25	CCCCAAACCCCATATGACAAG	ATTGCCCTCACATCGATCAG
MMP7	TTCCAAAGTGGTCACCTACAG	AGTTCCCCATACAACTTTCCTG
SERPINA3	CCTACAGACACCCAGAACATC	CCAAGGATAAGCAGACAGGG
FGF2	ACCCTCACATCAAGCTACAAC	ACTGGTGTATTTCCTTGACCG
CXCL1	AACCGAAGTCATAGCCACAC	CTGTGTCTCTCTTTCCTCTTCTG
CXCL10	TCTAAGTGGCATTCAAGGAGT	TGGCCTTCGATTCTGGATTC
CXCL16	CATCTTCATCCTCACCGCAG	GTATTAGAGTCAGGTGCCACAG
TNFSF12	TCGCAGCCCATTATGAAGTTC	GTGACTATAAACTCCCCGATCTG
NOD2	TCGTCAGCCAGTATGAATGTG	CAATCCATTCGCTTTCAGCG
SAA1	TTTTCTGCTCCTTGGTCCTG	TGGAAGTATTTGTCTGAGCCG
RUNX2	AGCAAGGTTCAACGATCTGAG	GGCGGTCAGAGAACAAACTAG
EREG	GAATATGTGGCTTTGACCGTG	TCCCTGCCCATAAGTTTGATG
JUN	AGCCCAAACTAACCTCACG	GTTACTGTAGCCATAAGGTCCG
Ki67	AAGTTCACACGGACGTCAG	GATGCTCTTGCCATCTCC
PCNA	GAGGCCTGCTGGGATATTAGC	GGGTGAGCTGCACCAAAGAG
MCM2	TTGGCGTGAGTTGCGTATTC	GAGACTGAAAACGATTACAAACATC
SNAI1	TCGGAAGCCTAACTACAGCGA	AGATGAGCATTGGCAGCGAG
SNAI2	ATGAGGAATCTGGCTGCTGT	CAGGAGAAAATGCCTTTGGA
CDH1	GTCACTGACACCAACGATAATCCT	TTTCAGTGTGGTGATTACGACGTTA
CDH2	CCATCAAGCCTGTGGGAATC	GCAGATCGGACCGGATACTG
CLDN1	TTGACTCCTTGCTGAATCTGAG	TTCTGCACCTCATCGTCTTC
CLDN7	GGTGGAGGCATAATTTTCATCG	ATGTTGGTAGGGATCAAAGGG
CYTK18	CAGAGACTGGAGCCATTACTT	GCCAGCTCTGTCTCATACTTG
ZEB1	AAGAAAGTGTTACAGATGCAGCTG	CCCTGGTAACACTGTCTGGTC
VIM	ACACCCTGCAATCTTTCAGACA	GATTCCACTTTGCGTTCAAGGT
TWIST	GCGCTGCGGAAGATCATC	GCTTGAGGGTCTGAATCTTGCT
ACTIN B	AGGCACCAGGGCGTGAT	GCCCACATAGGAATCCTTCTGAC
GAPDH	AATCCCATCACCATCTTCCAG	ATGACCCTTTTGGCTCCC
CCND1	TATTGCGCTGCTACCGTTGA	CCAATAGCAGCAAACAATGTGAAA
C-MYC	TCAAGAGGCGAACACACAAC	GGCCTTTTCATTGTTTTCCA
AXIN2	CGGGAGCCACACCCTTCT	TGGACACCTGCCAGTTTCTTT
COX2	CACAGTAGGAAGGTACCCAACACTATC	CTGCTAGAAAGGAGATCTGAGCCTGAG
CDH4	CTCATCTGCATCCTCATCCTG	CGCCTTCCTCGTCATACTTG
Ubr7	GAGAGATGGCTCAGTGGTTAAG	GAAGGCATGGACCTCATTACA
Gapdh	CTGGGTGGAGTGTCCTTTATC	GGTGAGACAGATTGTGAGGTAG

ChIP Primers

CDH4	GGACAAGGGAAAATAGGAAAAGG	TCCCAAATACCACACTCATGC
VDAC	TGAAAGGTGGAAAGAACTGGG	ATGGATATGTTAGAGCTGAAGTCTG
IARS	ACAGCTATTTTCTACGTCTGGAG	TGGATGCTCAGAGTTTTCGTC
GAPDH	TCCGGTGATGCTTTTCCTAG	TTTGCGGTGGAAATGTCCTTTTC

Supplementary Table 2: List of antibodies

Antibody	Company	Catalog Number	Dilution for WB	Dilution for IF
UBR7	Bethyl, Sigma	A304-130A, HPA000861	(1:500)	(1:200)
H2BK120Ub	CST, Milipore	5546, 05-132	(1:1000)	
H3K79Me2	Abcam	ab3594	NA	
E-Cadherin	CST	3195	(1:1000)	(1:100)
N-Cadherin	Novus	NBP1-48309	(1:2000)	(1:200)
Vimentin	Abcam	ab92547	(1:5000)	(1:1000)
Twist	Abcam	ab50887	(1:200)	
Ki67	Abcam	ab15580	(1:1000)	
PCNA	Abcam	ab92552	(1:1000)	
β-Catenin	Abcam	ab32572	(1:2000)	
GSK3β	Abcam	ab93926	(1:1000)	
Wnt3A	Abcam	ab81614	(1:500)	
β-TrCP	CST	4394	(1:1000)	
Axin2	CST	2151	(1:500)	
Cyclin D1	CST	2978	(1:500)	
C-Myc	CST	9402	(1:500)	
H3	Abcam	ab1791	(1:10000)	
H4	Abcam	ab10158	(1:1000)	
H2B	Abcam	ab1790, ab18977	(1:10000) (1:200)	
H2A	Abcam	ab18255	(1:5000)	
GST-HRP	GE HealthCare	RPN1236	(1:10000)	
Tubulin	Abcam	ab6046	(1:5000)	
GAPDH	Abcam	ab8245	(1:5000)	
B-Actin-HRP	Abcam	ab20272	(1:5000)	
Rabbit IgG HRP	Sigma	A1949	(1:10000)	
Mouse IgG HRP	Promega	W402B	(1:5000)	
Rabbit Alexa Fluor-488	Invitrogen	A11034		(1:1000)
Rabbit Alexa Fluor-564	Invitrogen	A11037		(1:1000)
Mouse Alexa Fluor-564	Invitrogen	A11032		(1:1000)
