# Supplementary material for: Atypical plant homeodomain of UBR7 functions as an H2BK120Ub ligase and breast tumor suppressor
Source: Nat Commun. 2019 Mar 28;10:1398. doi: 10.1038/s41467-019-08986-5 (PMC6438984; doi:10.1038/s41467-019-08986-5)
Supplement: Supplementary file 3 — Description of Additional Supplementary Files [file 41467_2019_8986_MOESM3_ESM.docx]

**Description of Additional Supplementary Files**

File Name: Supplementary Data 1

Description: List of H2BK120 peaks specific to MCF10A control or UBR7 knockdown cells.

File Name: Supplementary Data 2

Description: List of samples in tissue microarray.

File Name: Supplementary Data 3

Description: List of differentially expressed genes between control and UBR7 knockdown cells.

File Name: Supplementary Data 4

Description: GSEA analysis for differentially expressed genes between control and UBR7 knockdown cells.

File Name: Supplementary Data 5

Description: List of UBR7 targets and differentially expressed genes between control and UBR7 knockdown cells.

File Name: Supplementary Data 6

Description: GSEA analysis for UBR7 targets and differentially expressed genes between control and UBR7 knockdown cells.
