# Supplementary material for: Atypical plant homeodomain of UBR7 functions as an H2BK120Ub ligase and breast tumor suppressor
Source: Nat Commun. 2019 Mar 28;10:1398. doi: 10.1038/s41467-019-08986-5 (PMC6438984; doi:10.1038/s41467-019-08986-5)
Supplement: Supplementary file 10 — Reporting Summary [file 41467_2019_8986_MOESM10_ESM.pdf]

nature research

Corresponding author(s): Chandrima Das, Kunal Rai, Siddhartha Roy

Last updated by author(s): Jan 10, 2019

Reporting Summary

Nature Research wishes to improve the reproducibility of the work that we publish. This form provides structure for consistency and transparency in reporting. For further information on Nature Research policies, see Authors & Referees and the Editorial Policy Checklist.

Statistics

For all statistical analyses, confirm that the following items are present in the figure legend, table legend, main text, or Methods section.

n/a	Confirmed	
☐	☒	The exact sample size (*n*) for each experimental group/condition, given as a discrete number and unit of measurement
☐	☒	A statement on whether measurements were taken from distinct samples or whether the same sample was measured repeatedly
☐	☒	The statistical test(s) used AND whether they are one- or two-sided<br>*Only common tests should be described solely by name; describe more complex techniques in the Methods section.*
☒	☐	A description of all covariates tested
☒	☐	A description of any assumptions or corrections, such as tests of normality and adjustment for multiple comparisons
☐	☒	A full description of the statistical parameters including central tendency (e.g. means) or other basic estimates (e.g. regression coefficient) AND variation (e.g. standard deviation) or associated estimates of uncertainty (e.g. confidence intervals)
☐	☒	For null hypothesis testing, the test statistic (e.g. *F*, *t*, *r*) with confidence intervals, effect sizes, degrees of freedom and *P* value noted<br>*Give P values as exact values whenever suitable.*
☒	☐	For Bayesian analysis, information on the choice of priors and Markov chain Monte Carlo settings
☒	☐	For hierarchical and complex designs, identification of the appropriate level for tests and full reporting of outcomes
☒	☐	Estimates of effect sizes (e.g. Cohen's *d*, Pearson's *r*), indicating how they were calculated

Our web collection on statistics for biologists contains articles on many of the points above.

Software and code

Policy information about availability of computer code

Data collection	ChIP-seq: Raw fastq reads for all ChIP-seq experiments were processed using FastQC (http://www.bioinformatics.babraham.ac.uk/projects/fastqc/) and quality reads were aligned to the hg19 reference genome using Bowtie version 1.1.2 with the following criteria: -n 1 -m 1 --best --strata. Duplicate reads were marked using SAMBLASTER before compression to BAM files. To directly compare Control and UBR7-shRNA ChIP-seq samples, uniquely mapped reads for each mark were normalized by total reads per condition, sorted and indexed using samtools version 0.1.19.<br>RNA-seq: Raw FASTQ reads for all RNA-seq experiments were processed using FastQC and quality reads were aligned with the hg19 reference genome using TopHat(version 2.0.14) with a Bowtie2 (version 2.2.3) index based on UCSC annotations using the following criteria: -G -g 1 -r 150 --mate-std-dev 50 --library-type fr-unstranded.
Data analysis	Model-based analysis of ChIP-seq (MACS) (version 1.4.2; peak calling algorithm with a p-value threshold of 1e-7)54 was used to identify H2BK120Ub enrichment over “input” background. Unique H2BK120Ub binding sites were identified using the concatenate, cluster and subtract tools from the Galaxy/Cistrome web based platform.<br>Venn Diagrams were generated using the Venn Diagram tool in Galaxy. To visualize ChIP-seq libraries on the IGV browser we used deepTools version 2.4.060 to generate bigWig files by scaling the bam files to reads per kilobase per million (RPKM) using the following criteria: bamCoverage –b --normalizeUsing RPKM --smoothLength 300 --binSize 30 --extendReads 200 –o.<br>All read density plots were generated using thengs.plotpackage in R (version 3.3.1).<br>Chromatin State Calls:ChromHMM was used to identify combinatorial chromatin state patterns based on the histone modifications studied. Normalized bam files were converted into binarized data at a 1000bp resolution using the BinarizeBam command with a p-value cut-off of 1e-5. We specified that ChromHMM should learn a model based on 10 chromatin states. As we considered models between 8 and 20 chromatin states, we chose a 10-state model because it is large enough to identify important functional elements while still being small enough to interpret easily. Overlap Enrichment was used to compute differential enrichment in each of the 10-chromatin states between Control and UBR7-shRNA samples. The ChromHMM segment files from the 10-state model contain the genomic locations of each chromatin state called in both the Control and UBR7-shRNA samples. To determine which chromatin states were enriched between conditions we further compared the genomic locations by using the Control segments file as input for the segment directory, and by further separating the UBR7-shRNA segments file into 10 individual states and using it as input for the external coordinate directory. The

UBR7-shRNA segment file was separated into individual chromatin states for the external coordinate directory with the following command:
awk -F\t '{print >> $4;close($4)}' ~/path_to/UBR7-sh1_segments.bed
Overlap Enrichment was ran using the following command:
java -mx4000M -jar ChromHMM.jar OverlapEnrichment ~/path_to/Control_segments.bed ~/path_to/UBR7-shRNA_segments_separated OverlapEnrichment_Control_vs_UBR7
RNA-seq: To visualize RNA-seq libraries on the IGV browser we used deepTools version 2.4.060 to generate bigWig files by scaling the bam files to RPKM using the following criteria: bamCoverage –b --normalizeUsing RPKM --smoothLength 300 --binSize30 -o.
For identification of differentially expressed genes and Gene Set Enrichment Analysis (GSEA), raw counts were obtained by assigning reads at the gene level across the UCSC hg19 reference genome using featureCount in the Rsubread package. DESeq2 was employed for normalization and identification of differentially expressed genes in UBR7-shRNA and Control samples. All plots were generated using the ggplot and ggrepel packages in R. GSEA was run with normalized counts from all identified differentially expressed genes using the hallmark, curated and gene ontology gene-sets with default settings.

Statistical analyses pertaining to TMA were performed using R (version 3.3.1). All other statistical analyses were performed using Graphpad Prism (version 8).

For manuscripts utilizing custom algorithms or software that are central to the research but not yet described in published literature, software must be made available to editors/reviewers. We strongly encourage code deposition in a community repository (e.g. GitHub). See the Nature Research guidelines for submitting code & software for further information.

Data

Policy information about availability of data

All manuscripts must include a data availability statement. This statement should provide the following information, where applicable:
- Accession codes, unique identifiers, or web links for publicly available datasets
- A list of figures that have associated raw data
- A description of any restrictions on data availability

ChIP-Seq and RNA-Seq data can be accessed at GEO using the accession number: GSE93759 (https://www.ncbi.nlm.nih.gov/geo/query/acc.cgi?acc=GSE93759)

Field-specific reporting

Please select the one below that is the best fit for your research. If you are not sure, read the appropriate sections before making your selection.

☒ Life sciences ☐ Behavioural & social sciences ☐ Ecological, evolutionary & environmental sciences

For a reference copy of the document with all sections, see nature.com/documents/nr-reporting-summary-flat.pdf

Life sciences study design

All studies must disclose on these points even when the disclosure is negative.

Sample size	For in vivo experiments, sample size was determined by power analysis.<br>For all other experiments, sample size was determined on the basis of the magnitude and consistency of measurable differences between groups.
Data exclusions	All mice or samples were included in our analysis.
Replication	All the experimental findings were reliably reproduced.
Randomization	Animals are randomly allocated into the experimental groups.
Blinding	no blinding was done.

Reporting for specific materials, systems and methods

We require information from authors about some types of materials, experimental systems and methods used in many studies. Here, indicate whether each material, system or method listed is relevant to your study. If you are not sure if a list item applies to your research, read the appropriate section before selecting a response.

Materials & experimental systems

n/a	Involved in the study
☐	☒ Antibodies
☐	☒ Eukaryotic cell lines
☒	☐ Palaeontology
☐	☒ Animals and other organisms
☒	☐ Human research participants
☒	☐ Clinical data

Methods

n/a	Involved in the study
☐	☒ ChIP-seq
☒	☐ Flow cytometry
☒	☐ MRI-based neuroimaging

Antibodies

Antibodies used

Antibody, Company, Catalog Number
UBR7 Bethyl, A304-130A, Sigma HPA000861
H2BK120Ub CST, Milipore 5546, 05-132
H3K79Me2 Abcam ab3594
E-Cadherin CST 3195
N-Cadherin Novus NBP1-48309
Vimentin Abcam ab92547
Twist Abcam ab50887
Ki67 Abcam ab15580
PCNA Abcam ab92552
β-Catenin Abcam ab32572
GSK3β Abcam ab93926
Wnt3A Abcam ab8161
Axin2 CST 2151
Cyclin D1 CST 2978
C-Myc CST 9402
H3 Abcam ab1791
H4 Abcam ab10158
H2B Abcam ab1790, ab18977
H2A Abcam ab18255
GST-HRP GE HealthCareRPN1236
Tubulin Abcam ab6046
GAPDH Abcam ab8245
B-Actin-HRP Abcam ab20272
Rabbit IgG HRP Sigma A1949
Mouse IgG HRP Promega W402B
Rabbit Alexa Fluor-488 Invitrogen A11034
Rabbit Alexa Fluor-564 Invitrogen A11037
Mouse Alexa Fluor-564 Invitrogen A11032

Validation

All antibody validation statement is given on manufacturer's website.

Eukaryotic cell lines

Policy information about cell lines

Cell line source(s)

All cell lines were from MD Anderson Cancer Center Characterized Cell line core repository.

Authentication

All cell lines were validated by MD Anderson Cancer Center Characterized Cell line core facility via DNA fingerprinting.

Mycoplasma contamination

All cell lines used in the study were negative for mycoplasma.

Commonly misidentified lines
(See ICLAC register)

NA

Animals and other organisms

Policy information about studies involving animals; ARRIVE guidelines recommended for reporting animal research

Laboratory animals

Female nude mice aged 8-10 weeks (The Jackson Laboratory) were used for the experiments.

Wild animals

NA

Field-collected samples

NA

Ethics oversight

All mouse experiments were performed with the approval of the MD Anderson Institutional Animal Care and Use Committee.

Note that full information on the approval of the study protocol must also be provided in the manuscript.

ChIP-seq

Data deposition

☒ Confirm that both raw and final processed data have been deposited in a public database such as GEO.

☒ Confirm that you have deposited or provided access to graph files (e.g. BED files) for the called peaks.

Data access links<br>*May remain private before publication.*	GSE93759 (https://www.ncbi.nlm.nih.gov/geo/query/acc.cgi?acc=GSE93759)
Files in database submission	Control-H2BK120ub_ChIP-seq<br>Control-H3K4me1_ChIP-seq<br>Control-H3K9me3_ChIP-seq<br>Control-H3K27ac_ChIP-seq<br>Control-H3K79me2_ChIP-seq<br>Control-H3K4me3_ChIP-seq<br>Control-H3K27me3_ChIP-seq<br>Control-Input_ChIP-seq<br>UBR7-shRNA-H2BK120ub_ChIP-seq<br>UBR7-shRNA-H3K4me1_ChIP-seq<br>UBR7-shRNA-H3K9me3_ChIP-seq<br>UBR7-shRNA-H3K27ac_ChIP-seq<br>UBR7-shRNA-H3K79me2_ChIP-seq<br>UBR7-shRNA-H3K4me3_ChIP-seq<br>UBR7-shRNA-H3K27me3_ChIP-seq<br>UBR7-shRNA-Input_ChIP-seq
Genome browser session<br>(e.g. UCSC)	N/A

Methodology

Replicates	One replicate for each histone mark was performed for Control and shUBR7 samples.
Sequencing depth	Filename Total_QCpassed_Reads Duplicates Mapped Reduced_BAM<br>Control H2BK120ub 20731900 715412 13886885 13886885<br>Control H3K4me1 22668444 165450 16577379 16577379<br>Control H3K9me3 15379465 753101 9053474 9053474<br>Control H3K27ac 34889995 246777 24290500 22348499<br>Control H3K79me2 31821676 303100 22238696 22238696<br>Control H3K4me3 22625729 292682 16030371 16030371<br>Control H3K727me3 21897488 217064 16730639 13384501<br>Control Input 51514359 1289857 33713382 33713382<br>shUBR7 H2BK120ub 36260749 1044154 19405701 14165354<br>shUBR7 H3K4me1 40745902 265796 26679303 16541193<br>shUBR7 H3K9me3 25975890 1016726 13824775 9123544<br>shUBR7 H3K27ac 30545846 324859 22189899 22189899<br>shUBR7 H3K79me2 42379112 333315 26682713 22679721<br>shUBR7 H3K4me3 25750865 401166 15806451 15806451<br>shUBR7 H3K27me3 18718422 296205 13488130 13488130<br>shUBR7 Input 69719187 980595 45268020 33947449<br><br>All reads were of 36bp length.
Antibodies	H2BK120ub Millipore 17-650<br>H3K4me1 Abcam ab8895<br>H3K9me3 Abcam ab8898<br>H3K27ac Abcam ab4729<br>H3K79me2 Abcam ab3594<br>H3K4me3 Abcam ab8580<br>H3K27me3 Abcam ab6002
Peak calling parameters	Reads were aligned using Bowtie version 1.1.2 with the following criteria: bowtie -n 1 -m 1 --best --strata<br>Peaks were called using MACS version 1.4.2 with the following criteria: macs14 -t -c -f -g -p -n
Data quality	Control H2BK120ub = 8186/8426 peaks were over 5-fold and had an FDR less than 5%<br>UBR7 H2BK120ub = 1092/1205 peaks were over 5-fold and had an FDR less than 5%.
Software	Fastqc<br>Bowtie 1.1.2

Samtools 1.5
Deeptools 2.4
MACS 1.4.2
ChromHMM 1.7
Galaxy/Cistrome
Bedtools 2.24
ngsplot.r
IGV
